# Tailoring PES/PVAc Mixed Matrix Hollow Fiber Membranes with TiO_2_ Nanoparticles for Enhanced CO_2_/CH_4_ Separation Efficiency

**DOI:** 10.3390/polym18182236

**Published:** 2026-09-14

**Authors:** Tayyib Murtaza, Naveed Ramzan, Muhammad Saad Khan, Asif Jamil, Giedrius Janusas

**Affiliations:** 1 Department of Chemical Engineering, University of Engineering and Technology Lahore, Lahore 54890, Pakistan; tayyibbuttar@gmail.com (T.M.);; 2 Interdisciplinary Research Center for Membranes and Water Security, King Fahd University of Petroleum and Minerals, Dhahran 31261, Saudi Arabia; 3 Department of Mechanical Engineering, Faculty of Mechanical Engineering and Design, Kaunas University of Technology, LT-51424 Kaunas, Lithuania

**Keywords:** CO_2_ capture, methane enrichment, hollow fiber membranes, polyether sulfone, blend membrane

## Abstract

To use renewable energy and mitigate the greenhouse gas effect on the environment, carbon dioxide (CO_2_) separation is crucial. Recent research has shown that using membranes for CO_2_ separation is a vital option, but challenges remain with permeability and selectivity. Blend hollow fiber membranes offer a solution to these challenges. In this study, a blend of hollow fiber membranes made of polyether sulfone (PES) and polyvinyl acetate (PVAc) was developed, along with the incorporation of fillers, to overcome the permselectivity challenge. This resulted in the creation of mixed matrix blend hollow fiber membranes by using the phase inversion method. The membranes that were developed were examined using FESEM, FTIR, XRD, TGA, and pure-gas permeation analysis. The polymer blend demonstrated miscibility and preserved strong morphological and structural stability during CO_2_ and CH_4_ separation tests, exhibiting little deformation and reliable performance across different feed pressures ranging from 2 to 8 bar. The addition of TiO_2_ improved the compatibility of the polymer blend and increased the mobility of CO_2_. At 8 bar, the optimized PES/PVAc–5 wt.% TiO_2_ hollow fiber membrane reached a CO_2_ permeance of 92.22 GPU, representing an enhancement of about 24.7% in CO_2_/CH_4_ selectivity compared to the standard membrane. This emphasizes its potential for effective biogas upgrading under high pressure.

## 1. Introduction

Natural gas and biogas are important gaseous fuels that play a significant role in meeting the increasing global demand for cleaner and more sustainable energy [1]. While natural gas is widely utilized as a conventional energy source, biogas has gained considerable attention as a renewable alternative due to its potential to reduce greenhouse gas emissions and promote circular resource utilization [2]. However, both gas streams often contain substantial amounts of carbon dioxide (CO_2_), which lowers the calorific value of the fuel and reduces combustion efficiency [3]. Consequently, the selective separation of CO_2_ from methane (CH_4_) is a critical step in natural gas sweetening and biogas upgrading to produce methane-rich gas that satisfies pipeline, transportation, and industrial fuel specifications. Cryogenic distillation, liquid absorption, and solid adsorption are expensive ways to separate CO_2_ from CH_4_ due to high energy consumption, solvent regeneration, and equipment corrosion; compared to these methods, membranes provide an energy-efficient and scalable separation approach [4]. Membrane technology is used by a number of industries to separate CO_2_ from biogas and natural gas [5]. However, their effectiveness in separation largely depends on the nature of the polymeric material (glassy or rubbery), structure, as well as geometry of the membranes [6]. To solve this problem, researchers have tried many ways to modify the material (including chemical grafting, cross-linking, adding fillers (mixed matrix) and blends), structure (symmetric and asymmetric), and geometry of the membranes, like flat sheet, spiral wound, and hollow fiber membranes [7,8]. Polymer blending is a viable option to extend the performance of developed polymeric membrane materials. The performance of polymer blends is largely influenced by the miscibility between constituent polymers; based on the miscibility, the blends are classified as miscible blends, immiscible blends, and partially miscible blends. Each category exhibits different morphology and gas permeation behavior [9]. Blending of disparate polymers leads to new materials that can synergistically combine the properties of polymers that are not achievable by the individual component [10]. By varying the composition, specific characteristics are achieved for tailor-made applications.

The geometry of the membrane also plays a significant role in improving the trade-off between gas permeation and selectivity challenge. Hollow fiber membranes (HFMs), reported by many researchers, have been considered among the most practically feasible configurations in the application of gas separations due to their high surface-area-to-volume ratio, compact module design, and ability to fabricate ultra-thin selective skin layers compared with flat-sheet membranes [11,12]. In addition, HFMs provide better control over the morphology of membranes by adjusting the dope flow rate, bore fluid composition, and air gap distance [13]. These parameters play a critical role in governing membrane morphology and separation performance; therefore, achieving an optimal balance among them is essential to minimize structural defects and enhance overall membrane efficiency [14].

For previously reported blends of polyether sulfone (PES) with other glassy polymers, such as polyimide (PI), PES/PI blend membranes exhibited better compatibility, and the gas permeance of O_2_ reached values up to 210.8 GPU, while N_2_ transport increased with feed pressure [15]. Similarly, Khan et al. prepared blends such as SPEEK/Matrimid with cross-linking to resist plasticization, achieving PCO_2_ values of 9.43 Barrer, showing promise for CO_2_/CH_4_ separations under aggressive conditions [16]. Hadi et al. studied PES and polyvinyl acetate (PVAc) blends for CO_2_/CH_4_ separation; the membrane with 10 wt.% PVAc showed an increased CO_2_ permeance of 4.15 GPU, due to the acetate group in PVAc enhancing the solubility of CO_2_ within the membrane matrix [17]. Polymers containing oxygen-bearing functional groups, such as ether and carbonyl functional groups, are considered effective candidates for CO_2_ separation due to their favorable interactions with carbon dioxide molecules [18]. PVAc offers promising features, as its molecular structure includes both carbonyl and ester groups, which provide multiple interaction sites for CO_2_, potentially improving solubility and transport [19]. PES offers superior processability and the ability to form defect-free asymmetric hollow fibers compared with PEI (Ultem^®^) and PI (Matrimid^®^) under identical spinning conditions [20], while PI-based membranes required more stringent spinning conditions because of their rigid polymer chain structure [21]. Liang et al. compared the fillers and found that TiO_2_ provides better polymer–filler compatibility and higher CO_2_/CH_4_ selectivity than montmorillonite by reducing interfacial void formation [22]. TiO_2_ improves gas transport pathways, surface roughness, and CO_2_ interactions, thereby increasing permeability and selectivity [23]. N-methyl-2-pyrrolidone (NMP) was used as the solvent because comparative studies with DMF and DMAc demonstrated that it forms homogeneous polymer solutions, promotes controlled phase inversion, and produces uniform asymmetric membrane structures suitable for gas separation. Testing different loadings helps identify the optimal concentration that maximizes performance while minimizing agglomeration and structural defects [24]. A dope flow rate of 0.8 mL min^−1^ and a bore fluid flow rate of 1.0 mL min^−1^ were selected to maintain stable extrusion and balanced lumen formation, producing hollow fibers with uniform wall thickness and circular geometry; deviations from these flow rates have been reported to cause lumen deformation and dimensional instability [25]. An air gap of 10 cm was employed because it provides sufficient residence time for partial solvent evaporation and elongational stretching before coagulation, promoting the formation of a thin, defect-free selective layer while minimizing macrovoid formation and structural defects [26].

Previous studies on PES/PVAc membranes for CO_2_/CH_4_ separation have been limited to dense flat-sheet membranes. In this work, the novel fabrication of asymmetric PES/PVAc blend hollow fiber membranes (HFMs) is demonstrated, offering greater industrial relevance due to their high packing density, large membrane area, and excellent mechanical stability. Because defect-free polymer blend HFMs are highly sensitive to spinning conditions, their fabrication remains challenging. The effects of TiO_2_ nanoparticle incorporation on membrane morphology, filler dispersion, and CO_2_/CH_4_ separation performance are investigated, while the influence of filler loading and operating pressure is systematically evaluated. The fabricated blend HFMs have the potential for CO_2_/CH_4_ separation in natural gas sweetening and biogas upgrading under practical operating conditions.

## 2. Materials and Methods

### 2.1. Materials

The materials used in this study are discussed in Table 1. PES was used as the main polymer matrix. Polyvinyl acetate (PVAc) beads were used as the secondary polymer component for blending. The solvent used was N-methyl-2-pyrrolidone (NMP, 99.5% purity) for dissolving the polymers and preparing a homogenous dope solution. Titanium dioxide (TiO_2_) nanoparticles with an average particle size of 21 nm were incorporated as inorganic fillers.

### 2.2. Dope Solution Preparation

**Figure 1:**
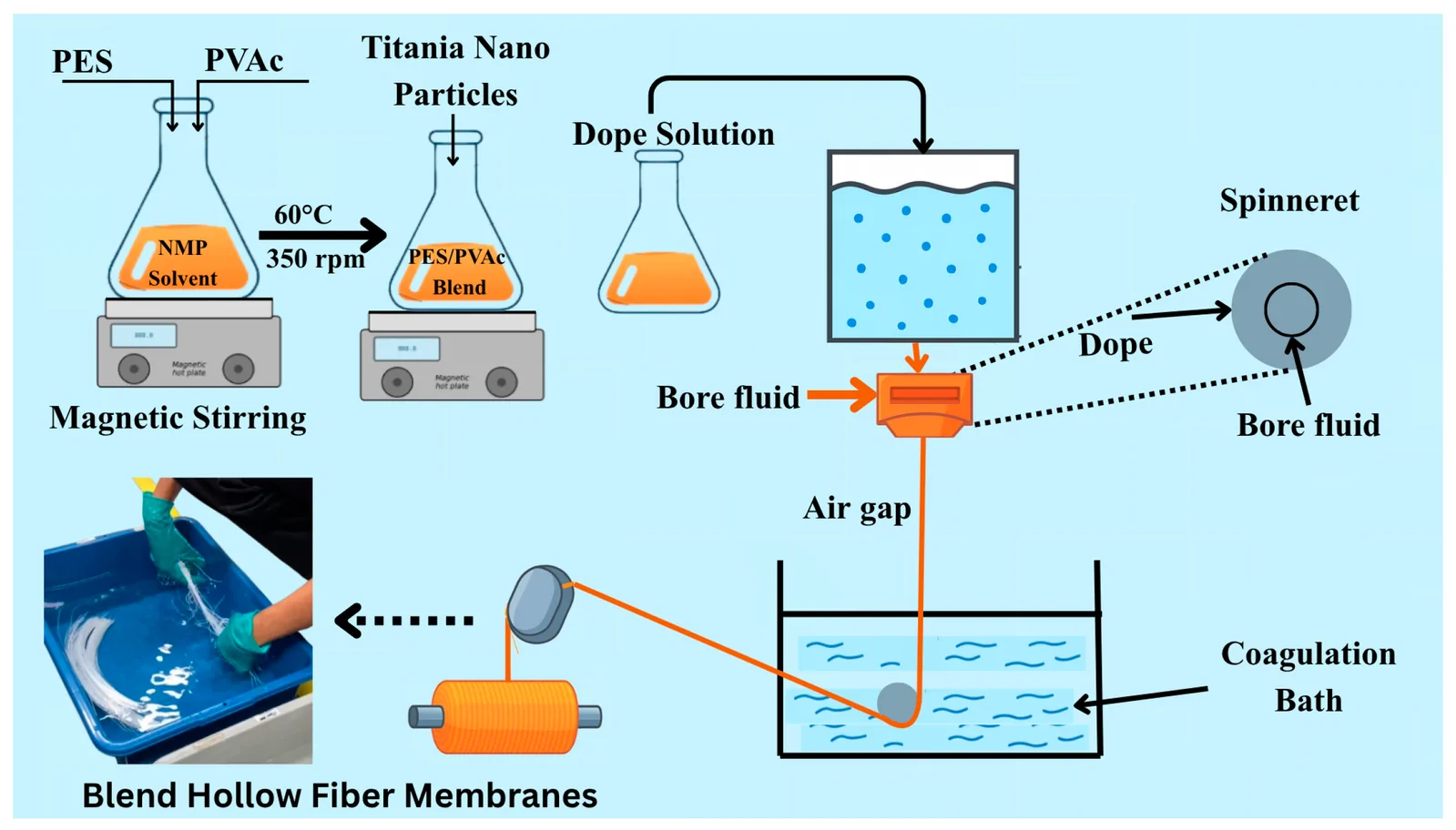
Schematic diagram of the hollow fiber membranes’ spinning process.

PES and PVAc were dried at 60 °C for 24 h before solution preparation to remove residual moisture in a vacuum oven (Model: 52 L vacuum oven, RT + 10–250 °C, 133 Pa). The total polymer concentration in all dope solutions was set at 20 wt.%, with a constant blend ratio of 90:10 (PES:PVAc) based on weight. The composition of the various dope solutions prepared is discussed in Table 2. First, PES was added to NMP and stirred on a magnetic hot plate (IKA, C-MAG HS7, Staufen, Germany) at room temperature to initiate dissolution. Then, PVAc was added slowly with continuous mixing once PES was partially dissolved [17]. Nanoparticles were added to the polymer solution after complete dissolution and dispersed thoroughly at 60 °C to ensure proper distribution within the matrix [27]. Each dope solution was stirred at 350 rpm for 24 h, degassed under vacuum for 30 min, and left to stand at room temperature for 2 h prior to membrane spinning [28]. Figure 1 shows a schematic representation of the developed blend HFMs.

### 2.3. Blend Hollow Fiber Membrane Development

HFMs composed of PES/PVAc and titania nanoparticles were fabricated using the dry–wet phase inversion technique on a laboratory-scale spinning setup [29]. A stainless-steel spinneret with an outer diameter of 0.8 mm and an inner diameter of 0.4 mm was employed for extrusion. The polymer dope solution was delivered through the spinneret at a constant rate of 0.8 mL/min using nitrogen gas at a pressure of 1 bar. Simultaneously, tap water was used as the bore fluid and introduced at a flow rate of 1.0 mL/min using a syringe pump. An air gap of 10 cm was maintained between the spinneret and the surface of the coagulation bath to allow partial solvent evaporation. The extruded fibers entered a water coagulation bath kept at room temperature, where phase separation occurred. The resulting fibers were collected under gravity and submerged in fresh water for 72 h to remove residual solvent. The water was replaced every 24 h. After solvent removal, the fibers were air-dried at room temperature before further testing and characterization.

### 2.4. Blend HFMs Characterization

Scanning electron microscopy (SEM) examination with the Nova Nano SEM 450 (Thermo Fisher Scientific, Hillsboro, OR, USA) was employed to investigate the morphology of the HFMs. Fourier-transform infrared spectroscopy (FTIR) (Perkin Elmer 1650, PerkinElmer, Waltham, MA, USA) determined the functional groups attached to the synthesized membranes. The spectra of each membrane were obtained in the 400–4000 cm^−1^ wavenumber region using the transmission mode. To evaluate the thermal stability of PES MMMs, thermogravimetric analysis (TGA, STA6000, PerkinElmer, Waltham, MA, USA) was employed. The heating rate was maintained at 10 °C min^−1^ across a temperature range of 60 °C to 800 °C, and the thermal behavior was analyzed in a nitrogen atmosphere. For X-ray diffraction (XRD) analysis of blend HFMs; measurements were performed using a Bruker D8 Advance diffractometer (Bruker AXS GmbH, Karlsruhe, Germany) equipped with Cu Kα radiation (λ = 1.5406 Å) operating at 40 kV and 40 mA, scanned over a 2θ range of 5° to 80° with a step size of 0.02°.

The gas permeation performance of PES/PVAc-titania HFMs was evaluated using high-purity (99.999%) carbon dioxide (CO_2_) and methane (CH_4_) at room temperature, across a pressure range of 2 to 8 bar. During testing, the desired gas was introduced through the lumen side of the fiber bundle, and a soap bubble flow meter was deployed to find permeation [30].

For each test, six hollow fibers were bundled together, and one end of the bundle was sealed using epoxy resin. After an initial curing period of 3 h, a second coat of epoxy was applied, followed by 24 h of drying to ensure complete sealing. Membrane permeance and selectivity for CO_2_ and CH_4_ were calculated by using Equations (1) and (2), respectively [31].

(1) $$\frac{P_{i}}{l}=\frac{Q_{i}}{T_{i}}\frac{273}{A\Delta p_{i}}$$

(2) α=PCo2∕lPCH4l

Q = volumetric flow rate (cm^3^/s), T = temperature (K), A = effective area of the membrane (cm^2^) $=n\pi DL_{m}$, (n = number of fibers, D = outer diameter, L_m_ = effective length of fiber) $\Delta p_{i}$ = trans membrane pressure (cm. Hg), l  = membrane thickness (cm). α is the selectivity.

## 3. Results and Discussion

### 3.1. Chemical Analysis

**Figure 2:**
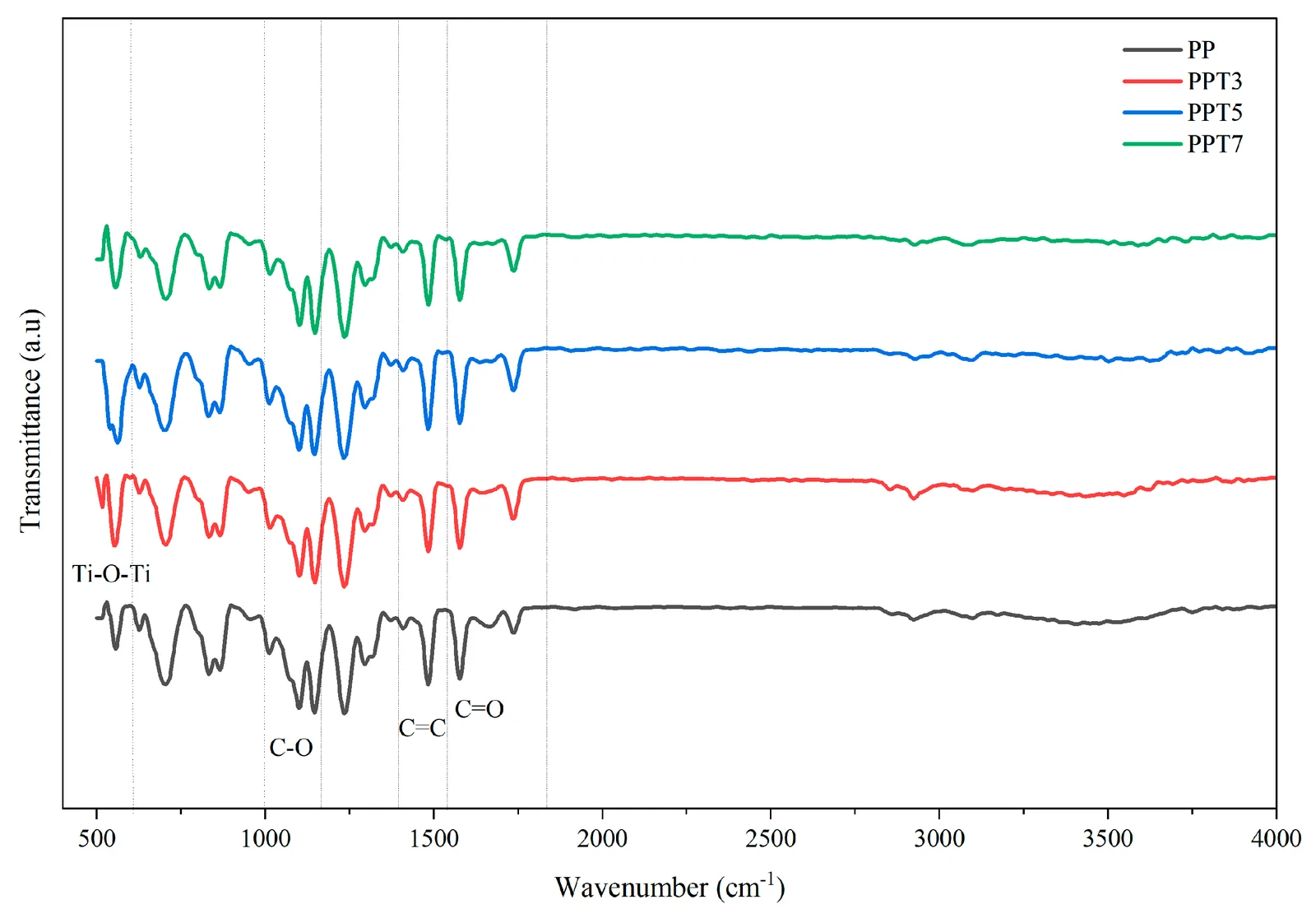
FTIR analysis of developed blend HFMs.

Chemical structure and interactions among PES, PVAc, and TiO_2_ nanoparticles in the fabricated membranes were characterized using Fourier-transform infrared spectroscopy (FTIR) and shown in Figure 2. In this study, FTIR analysis was performed to identify characteristic functional groups and assess possible chemical interactions between the polymer blend and TiO_2_. FTIR spectra were recorded to confirm the presence of functional groups in PES, PVAc, and any possible interactions between them.

Table 3 discusses all the characteristic peaks of PES/PVAc and the incorporation of TiO_2_, and the peaks were observed at 1150 cm^−1^, corresponding to –SO_2_– symmetric and asymmetric stretching, and aromatic ring vibrations. PVAc showed prominent peaks around 1735 cm^−1^ attributed to C=O stretching from the acetate group [32]. FTIR peaks are well in accordance with the literature [17,27,33]. The broad infrared absorption spanning 900–500 cm^−1^ originates from the characteristic lattice vibrations of the Ti–O bonds and the O–Ti–O network [34]. Previous work by Tutuk et al. on the PES-TiO_2_ coated with PVA has also demonstrated the peaks associated with PES and TiO_2_ [35].

### 3.2. Structural (Phase and Crystallinity) Characterization

**Figure 3:**
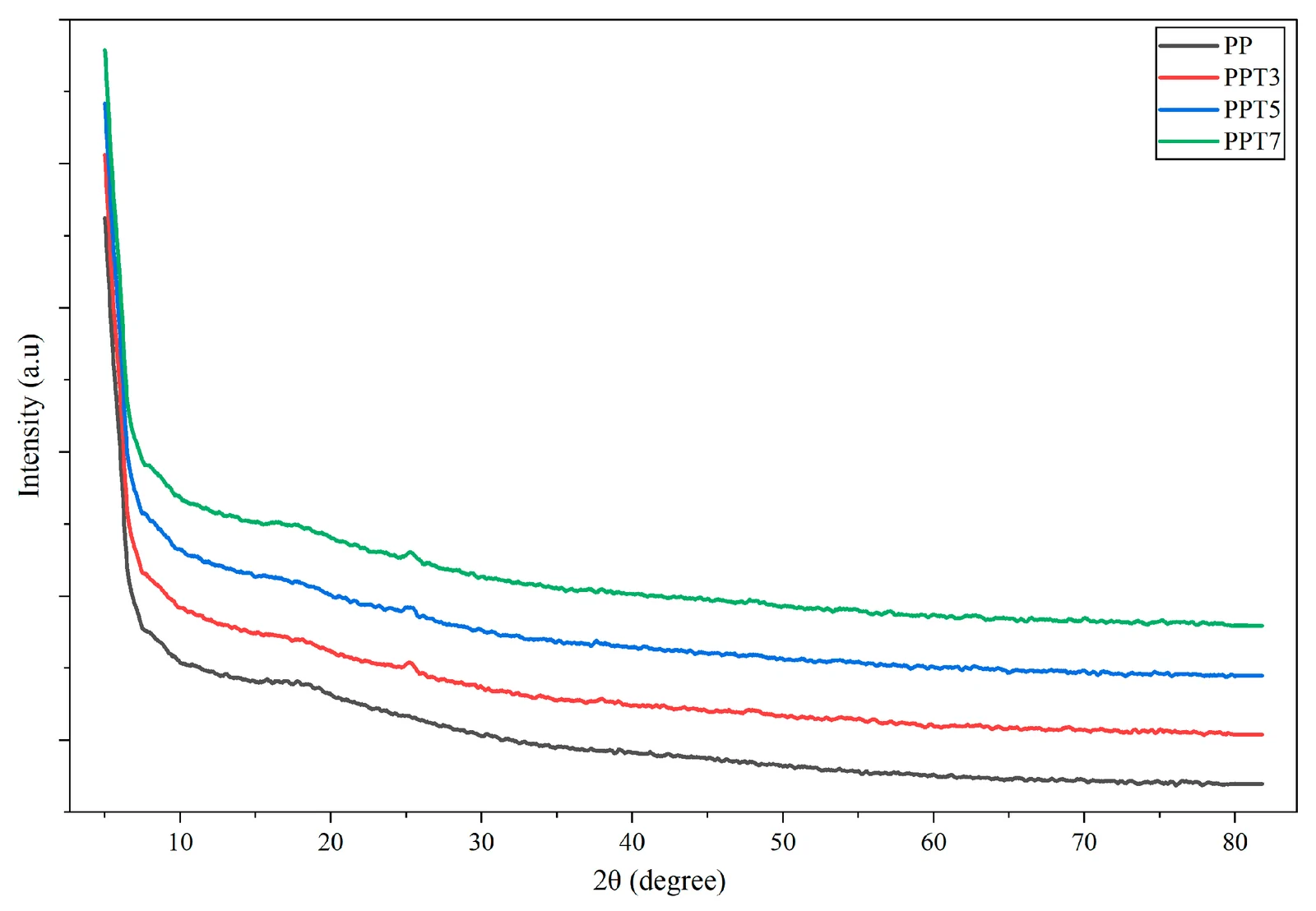
XRD pattern of PES/PVAc and PES/PVAc-TiO_2_ HFMMM.

Figure 3 provides the XRD pattern of the neat PES/PVAc membrane showed a broad halo around 2θ ≈ 17–22°, confirming its predominantly amorphous structure as depicted in Figure 3. Upon incorporation of 21 nm TiO_2_ nanoparticles, weak diffraction peaks appeared at 25.3°, 37.8°, and 48.0°, corresponding to the (101), (004), and (200) crystal planes of anatase TiO_2_. The polymer background remained amorphous [40], and no secondary phases or degradation-related peaks were observed [34]. The peaks are in agreement with JCPDS Card No. mentioned in Table 4, and the findings also align with the research conducted by Nidhi et al. regarding the XRD patterns of PES/TiO_2_ HFMs [44].

The TiO_2_ diffraction peaks appeared with relatively low intensity, which is attributed to the low concentration of nanoparticles dispersed within the predominantly amorphous polymer matrix. This is also confirmed by the previously published data on PES-TiO_2_ [35].

### 3.3. Thermal Analysis

**Figure 4:**
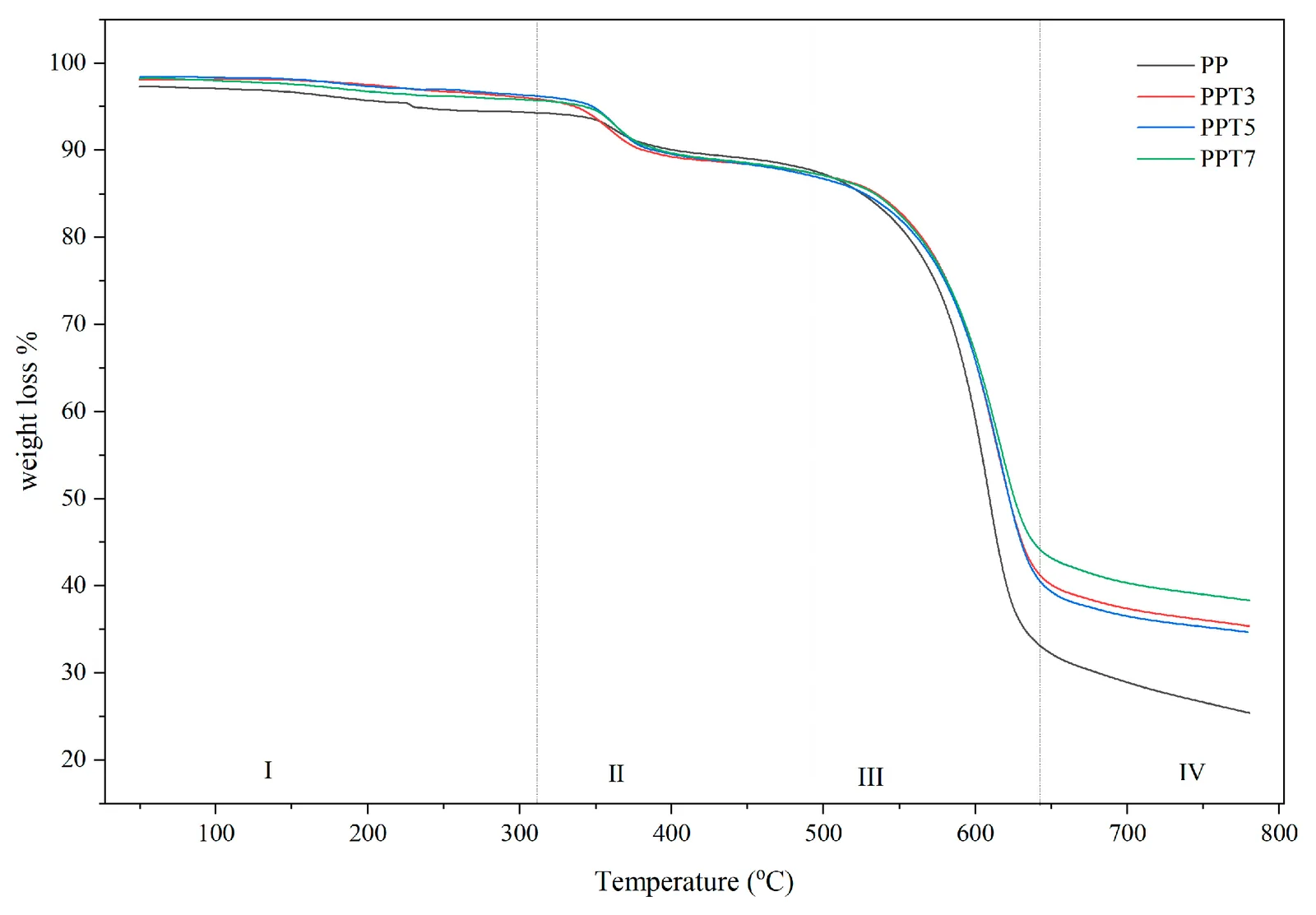
TGA of PES/PVAc and PES/PVAc-TiO_2_ HFMMM.

Figure 4 shows the TGA of the blend HFMs, performed to assess the thermal behavior of the blend HFMs and to see how the addition of TiO_2_ nanoparticles would affect their degradation behavior. No noticeable weight loss was observed below 100 °C, indicating that the membranes were free from residual moisture and volatile components. The degradation occurred in multiple stages, where the initial weight loss was associated with the decomposition of the PVAc phase, followed by the main degradation of the PES matrix at higher temperatures. The first degradation stage is attributed to the deacetylation of the PVAc component, during which acetate side groups are eliminated, producing acetic acid and leaving unsaturated polyene sequences along the polymer backbone [45]. At higher temperatures, the main degradation stage corresponds to the cleavage of the PES backbone through the scission of aryl ether (–O–) and sulfone (–SO_2_–) linkages, resulting in the formation of low-molecular-weight aromatic compounds and volatile degradation products [22,46].

The incorporation of TiO_2_ influenced the thermal degradation behavior of the membranes. The T_5_ value increased from 229.74 °C for the PP membrane to 336.41 °C, 348.01 °C, and 342.59 °C for the PPT3, PPT5, and PPT7 membranes, respectively. Likewise, the T_50_ value increased from 609.31 °C for PP to 622.25–625.70 °C for the TiO_2_-containing membranes, indicating a delay in the degradation process.

The residual mass also increased with TiO_2_ loading, from 25.38% for the PP membrane to 35.36%, 34.66%, and 38.30% for the PPT3, PPT5, and PPT7 membranes, respectively, as shown in Table 5. The shift in degradation temperatures indicates that TiO_2_ delayed the thermal degradation process, which has been attributed to the barrier effect of well-dispersed nanoparticles that restrict polymer chain mobility and reduce the diffusion of volatile degradation products.

### 3.4. Morphological Analysis

**Figure 5:**
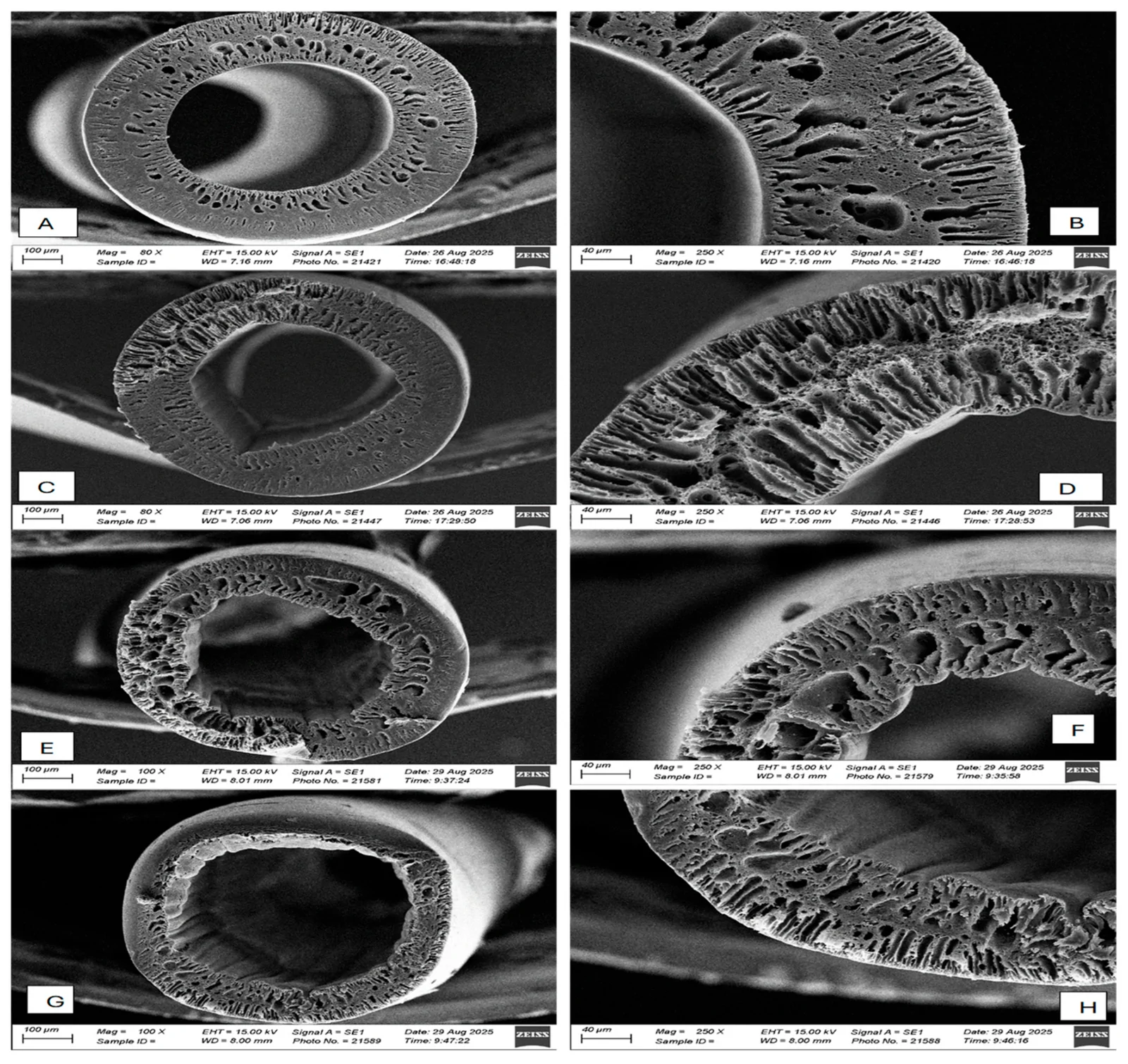
Cross-sectional SEM images of PES/PVAc (**A**,**B**), PES/PVAc with 3 wt.% TiO_2_ (**C**,**D**), PES/PVAc with 5 wt.% TiO_2_ (**E**,**F**), and PES/PVAc with 7 wt.% TiO_2_ (**G**,**H**).

Prior to analysis, the blend HFM specimens were cryo-fractured in liquid nitrogen, dried, and mounted onto aluminum stubs with conductive carbon tape; the samples were sputter-coated with gold for 60 s (15 mA) using a JEOL JFC-1600 auto fine coater (Tokyo, Japan). SEM images of the fabricated membranes clearly showed the influence of the content of TiO_2_ on the internal structure. PES/PVAc membranes showed well-developed finger-like macrovoids extending from the dense skin layer toward the lumen side. This behavior is attributed to the instantaneous demixing process during immersion in the non-solvent bath, which accelerates the solvent–non-solvent exchange during phase inversion, promoting rapid demixing and elongated pore growth. When the TiO_2_ concentration increased to 3 wt.%, SEM cross-sectional images Figure 5C,D showed that the finger-like macrovoids became more pronounced and extended deeper from the outer skin toward the lumen. This effect can be attributed to the hydrophilic nature of TiO_2_ nanoparticles, which accelerated solvent–non-solvent exchange during phase inversion, thereby promoting the growth of elongated macrovoids. The reduction in macrovoids can be explained by the increased viscosity of the casting solution, as well as stronger interactions between TiO_2_ and the polymer chains, which slow down phase separation. This balance between demixing kinetics and dope viscosity resulted in membranes with a more homogeneous porous network and improved polymer–filler adhesion.

At higher loadings of 7 wt.% TiO_2_, the SEM analysis in Figure 5G,H revealed nanoparticle agglomeration within the polymer matrix. These aggregates disrupted the continuity of the structure and caused partial collapse of finger-like pores. Moreover, the formation of irregular interfacial voids around the TiO_2_ clusters created unselective pathways for gas transport, which could undermine the intended improvement in separation performance. Such effects are consistent with earlier studies reporting that excessive nanoparticle incorporation can lead to poor dispersion and weak interfacial interactions [47]. At 3 wt.% TiO_2_, the morphology displayed optimum features, with well-dispersed nanoparticles embedded in the matrix, consistent with reports highlighting that moderate loadings enhance interfacial compatibility and improve structural compactness without sacrificing porosity. However, at higher loading (7 wt.%), SEM revealed particle agglomeration, surface roughness, and the formation of interfacial voids that compromise membrane integrity. This result aligns with previous studies on PES–TiO_2_ MMMs, which indicated that elevated filler content led to nanoparticle aggregation and defect formation, hence compromising integrity and reducing selectivity [33]. According to the literature, PES membranes filled with silica and zeolite tend to have bigger gaps between the two sides when they are loaded the same way.

**Figure 6:**
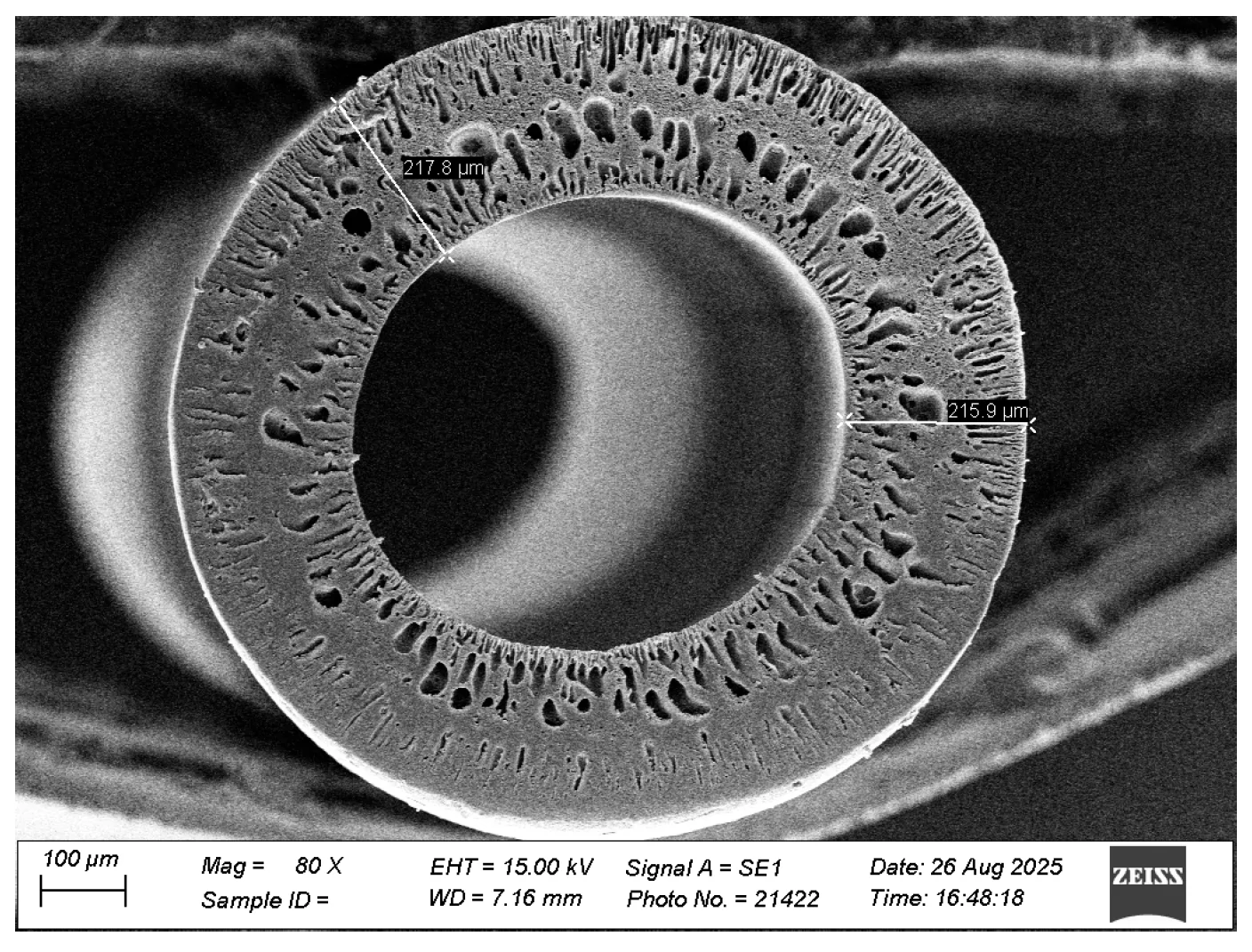
Thickness of blend HFMs.

In PES/PVAc–TiO_2_ asymmetric hollow fiber membranes with an overall wall thickness of ~216 µm, as shown in Figure 6, gas separation is governed by the dense selective skin layer, which controls CO_2_/CH_4_ transport through differences in solubility and diffusivity [48]. The addition of TiO_2_ and PVAc modifies phase inversion behavior and enhances surface polarity, which can improve CO_2_ affinity and influence skin layer formation. The porous substructure accounts for most of the thickness and provides mechanical support with minimal resistance to gas transport, making the integrity of the selective skin layer the key determinant of performance.

### 3.5. Effect of Feed Pressure on Gas Permeation

**Figure 7:**
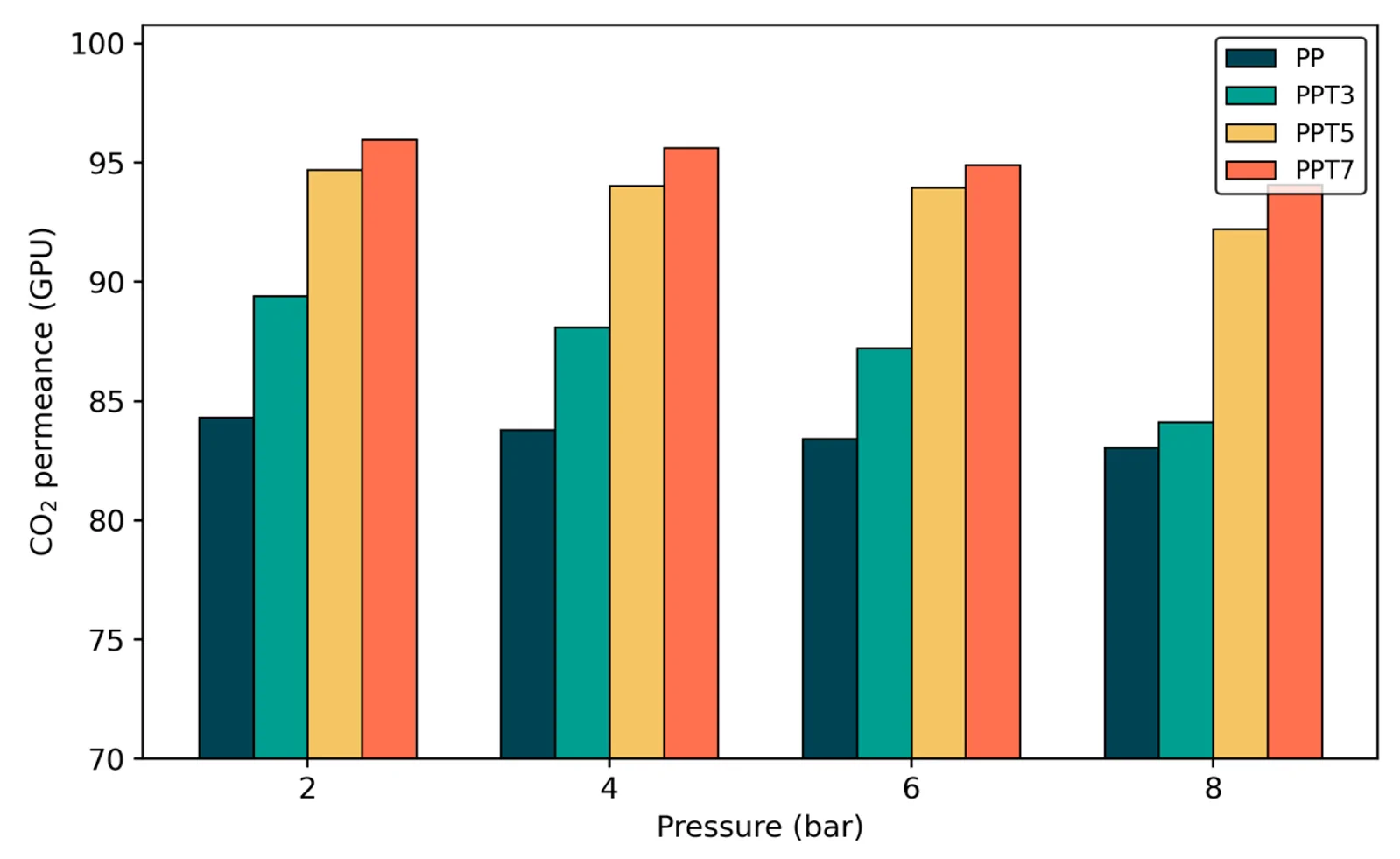
CO_2_ permeation vs. pressure of PES/PVAc and PES/PVAc-TiO_2_ HFMMM.

**Figure 8:**
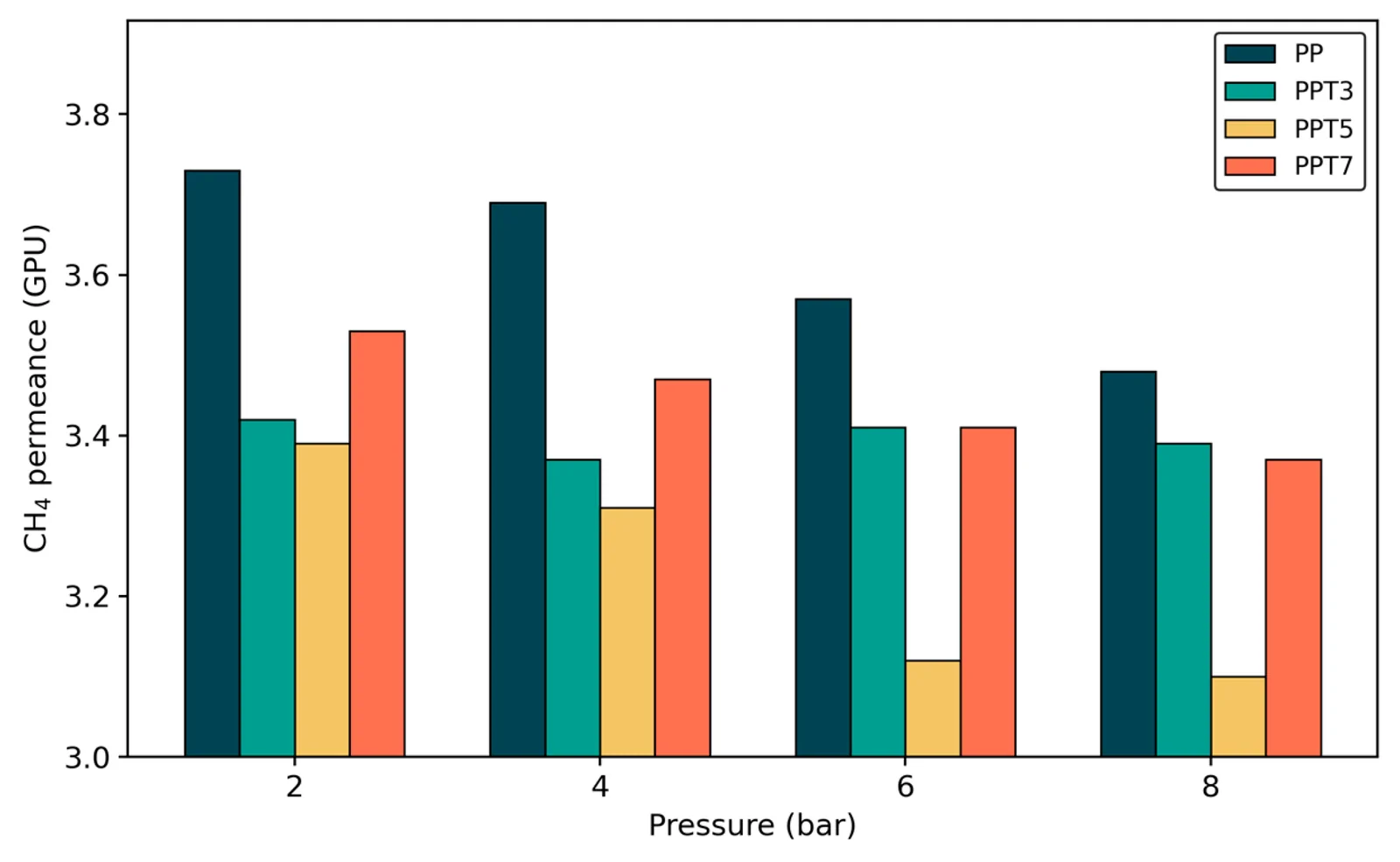
CH_4_ permeation vs. pressure of PES/PVAc and PES/PVAc-TiO_2_ HFMMM.

The effect of pressure on the flow of CO_2_ and CH_4_ across PES/PVAc and TiO_2_-modified mixed membranes at pressures ranging from 2 to 8 bar is shown in Figure 7 and Figure 8. It illustrates that for all membrane compositions, the permeabilities of both CO_2_ and CH_4_ consistently decreased as pressure increased. With increased pressure on the polymer chains, they compact, thereby limiting the ability of the gas to disperse because of less spatial availability of space.

Figure 7 shows the CO_2_ permeation at different pressures ranging from 2 to 8 bar in GPU. PES/PVAc HFMs permeance is 84.32 GPU at 2 bar, and with the addition of titania fillers for 3 wt.%, this value increases to 89.41 GPU; with 5 and 7 wt.% titania loading, it further increases to 94.71 and 95.97 GPU, respectively. As shown by the gas permeation values, it is obvious that well-dispersed titania nanoparticles provide extra passages for the flow of gases, which helps to increase the permeability by 6.5% as compared to pure PES/PVAc blend hollow fiber membranes. With an increase in feed pressure from 2 to 8 bar, the permeability values decrease by a margin, but the membranes keep their capacity at 97% for the CO_2_ permeability.

The optimal PES/PVAc membrane’s permeance went down from 84.32 GPU at 2 bar to 83.06 GPU at 8 bar. On the other hand, the membranes made of TiO_2_ let more CO_2_ through at all pressures. At 2 bar, the 5 wt.% TiO_2_ membrane had a permeance of 94.71 GPU, while at 8 bar, it had a permeance of 92.22 GPU. TiO_2_ nanoparticles have hydroxyl groups all over their surfaces, which interact strongly with CO_2_ molecules. This makes it easier for CO_2_ to dissolve and spread out. Well-dispersed nanoparticles also provide more free space and microchannels at the polymer–filler interface, which makes it easier for CO_2_ to pass through. This keeps the polymer matrix from being too thick and lets CO_2_ pass through even while it is under pressure. PVAc adds polar acetate groups that make CO_2_ more soluble, while PES gives the mixture mechanical stability and makes it less likely to compress under pressure.

**Figure 9:**
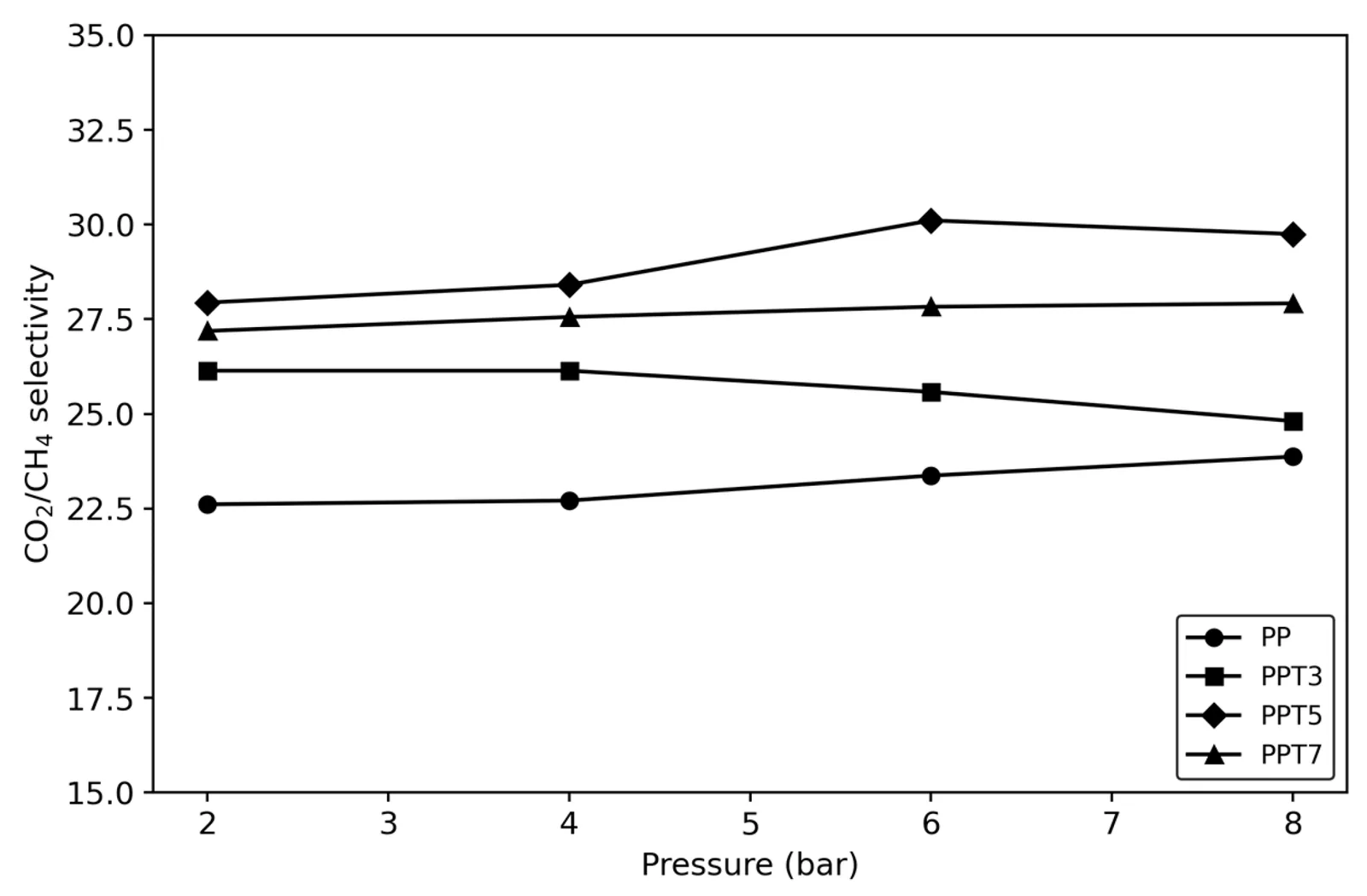
Selectivity CO_2_/CH_4_ of PES/PVAc and PES/PVAc-TiO_2_ HFMMM.

Adding TiO_2_ to the membranes made them less permeable to CH_4_ because the filler particles made the interfacial areas stiffer, which made the diffusion route longer. This alteration in structure makes it tougher for CH_4_ to get through, which makes the CO_2_/CH_4_ selectivity better, as shown in Figure 9. How effectively the two gases may flow through the membrane at the same time determines the overall CO_2_/CH_4_ selectivity. An incremental increase in membrane selectivity was observed with increasing pressure until an optimal operating range of approximately 4–6 bar was achieved, as shown in Figure 9. After that, it remained the same or dropped down when the pressure was higher (8 bar). CO_2_ is superior because its molecules are smaller (3.3 Å instead of 3.8 Å for CH_4_).

Further performance improvement was achieved by the addition of TiO_2_ nanoparticles. The addition of 5 wt% and 7 wt% TiO_2_ increased CO_2_ permeability to a range of 89–96 GPU, while the selectivity also improved to 25–30 within the tested pressure range. These results represent significant enhancement both in permeability and selectivity compared to the unfilled PES/PVAc blend. The presence of TiO_2_ would have favored CO_2_ sorption due to its surface hydroxyl groups and extra transport channels at the interfacial region of the polymer–filler, which enhanced the gas diffusion without affecting selectivity. The improvement indicates better filler interaction within the polymer matrix.

When compared with other reported polymeric and blend systems—such as PEI/MMT [49], PSf/PEG [50], and PSf/ZIF-8 [51]—the developed membranes have competitive or higher CO_2_ permeability with comparable selectivity. Unlike in some mixed matrix membranes where filler agglomeration or plasticization reduces performance at higher filler loadings, the TiO_2_-based membranes maintained good mechanical integrity and stable performance up to 8 bar.

In comparison with traditional glassy polymers such as Matrimid^®^ 5218 and PEI, which generally exhibit CO_2_ permeability below 30 GPU [52], the present membranes achieved almost double the permeability while maintaining similar selectivity levels. Although rubbery polymers such as Pebax^®^ 1657 show much higher permeability (110–130 GPU), their selectivity remains lower (≈9–10,) [53]. K. Zahri et al. prepared a graphene oxide/polysulfone hollow fiber mixed matrix membrane for gas separation in their work. The gas permeation improved as compared to the nascent polysulfone membrane due to the addition of 0.25% graphene oxide by 14%, and CO_2_ permeance reached 74.47 GPU, while CH_4_ permeance decreased from 3.90 to 2.63 GPU at 5 bar [54]. The reduction in CH_4_ permeance and the increment in CO_2_ value relate to the nature of gases like CO_2_, which is highly condensable, with polymer ideal solubility of 29.5 × 10^−3^ cm^3^ (STP)/cm^3^ cm Hg, and CH_4_ with low solubility; the increment in CO_2_ permeance is also due to the strong affinity of graphene oxide towards CO_2_. In our work, a similar trend is observed with permeation value increments due to the ideal solubility and structure of the membrane skin layer. Due to the defects on nascent blend hollow fiber membranes, more gases are allowed to pass through the undesired voids, but the addition of nanoparticles—thinner, denser, and defect-free skin layers—restricted the permeation of nonselective gases such as CH_4,_ as shown in Figure 8, and improved the selectivity of blend HFMs. The current system therefore provides a favorable balance between permeability and selectivity, approaching the upper bound for polymeric membranes used in CO_2_/CH_4_ separation.

The values provided in Table 6 are average values of three experiments. The neat blend membranes’ selectivity is 22.605. Table 6 provides a brief comparison of the developed PES/PVAc and the incorporation of titania MMM HFMs with previously published data. PES/PVAc/TiO_2_ membranes under pressures ranging from 2 bar to 8 bar revealed exceptional mechanical strength and separation efficiency. The hybrid functionality of TiO_2_ nanoparticles, which create rigid molecular sieving channels, along with the dense, selective structure formed by the PVAc blend, effectively inhibited matrix swelling at increased pressures. The ideal selectivity increased at a feed pressure of 2 bar; the PES/PVAc membrane demonstrated a CO_2_/CH_4_ selectivity of 22.61. The addition of 3 wt.% TiO_2_ raised the selectivity to 26.14, reflecting a 15.65% improvement, which suggests that well-dispersed TiO_2_ nanoparticles enhanced the interfacial free volume while maintaining the membrane’s molecular sieving abilities. Increasing the TiO_2_ content to 5 wt.% further boosted the selectivity to 27.94, marking the highest enhancement of 23.59% compared to the pure membrane. This indicates that this level of TiO_2_ loading optimizes the interaction between the polymer and the filler while reducing nonselective transport pathways. However, raising the TiO_2_ concentration to 7 wt.% led to a selectivity of 27.19, which, while still 20.27% greater than that of the unmodified membrane, was 2.69% lower than the selectivity observed at 5 wt.%. Overall, the improvement in gas transport performance can be linked to the synergistic interaction between polymer blending and nanoparticle incorporation [55], which modifies membrane morphology, increases free volume, and introduces CO_2_-philic pathways. The PES/PVAc–TiO_2_ hollow fiber membranes combine good gas separation performance, stability, and scalability, making them suitable for biogas upgrading and CO_2_ capture applications.

## 4. Conclusions

PES/PVAc blend HFMs were successfully formed with various TiO_2_ nanoparticle loadings (3, 5, and 7 wt.%) for CO_2_/CH_4_ gas separation. The tailored blend HFMs exhibited structural and operational stability, effectively resisting performance degradation and structural deterioration even under high feed pressures, achieving a stable CO_2_ permeance ranging from 83 to 94 GPU across the investigated pressure range. The presence of carbonyl oxygen and ester oxygen in PVAc, along with ether oxygen in the PES backbone, was confirmed by FTIR analysis. The characteristic finger-like membrane structure was verified by SEM images, while XRD analysis revealed the characteristic diffraction peaks corresponding to TiO_2_ nanoparticles. The thermal stability of the membranes was further improved, as confirmed by TGA analysis. Among all fabricated membranes, the PES/PVAc membrane containing 5 wt.% TiO_2_ (PPT5) exhibited the best gas separation performance, achieving a CO_2_ permeance of 92.22 GPU with a CO_2_/CH_4_ selectivity of 29.74 at 8 bar.

## Figures and Tables

**Table 1:** Materials used in this study.

Material	Identifier	Supplier
PES	ULTRASON^®^ E-6020 P Mw ≈ 50,000 g/mol; Repeat unit: (C_27_H_22_O_4_S)_n_	BASF, Ludwigshafen, Germany
PVAc	Mw = 100,000 g/mol; Repeat unit: (C_4_H_6_O_2_)_n_	Sigma-Aldrich, St. Louis, MO, USA
NMP	Purity: 99.5%; Formula: C_5_H_9_NO	Sigma-Aldrich, St. Louis, MO, USA
TiO_2_	Average particle size: 21 nm; Formula: TiO_2_	Sigma-Aldrich, St. Louis, MO, USA

**Table 2:** Dope solution composition.

Code	HFMs	PES (wt.%)	PVAc (wt.%)	TiO_2_ (wt.% of Polymer)
PP	PES/PVAc	18.0	2.0	0.0
PPT3	PES/PVAc + 3 wt.% TiO_2_	18.0	2.0	3.0
PPT5	PES/PVAc + 5 wt.% TiO_2_	18.0	2.0	5.0
PPT7	PES/PVAc + 7 wt.% TiO_2_	18.0	2.0	7.0

**Table 3:** Characteristic peaks of the developed blend HFMs in the nascent state and with the incorporation of TiO_2_.

Sr. No.	Functional Group	Bond Assignment	Theoretical Range (cm^−1^)	PP cm^−1^	PPT3 cm^−1^	PPT5 cm^−1^	PPT7 cm^−1^	Interpretation	Ref.
1	O-H stretching	Surface hydroxyl (TiO_2_ and adsorbed water)	3200–3600	3421	3416	3412	3407	Hydrogen bonding between TiO_2_-OH and polymer chain	[36]
2	C-H stretching	-CH_2_/-CH_3_	2850–2960	2923	2922	2921	2920	Polymer backbone remained unchanged	[37]
3	C=O stretching	PVAc carbonyl	1730–1750	1736	1734	1732	1731	Hydrogen bonding between C=O and TiO_2_ surface -OH	[38]
4	Aromatic C=C stretching	PES benzene ring	1480–1600	1583	1582	1581	1580	Aromatic structure maintained	[39]
5	O=S=O asymmetric stretching	PES sulfone	1290–1330	1321	1319	1318	1317	Physical interaction between sulfone group and TiO_2_	[40]
6	Ar-O-Ar stretching	PES ether linkage	1220–1250	1239	1238	1237	1236	Polymer chain integrity retained	[41]
7	O=S=O symmetric stretching	PES sulfone	1120–1160	1149	1148	1146	1145	Slight interaction with TiO_2_	[42]
8	C-O stretching	PVAc	1020–1070	1044	1043	1042	1041	Interaction between PVAc and TiO_2_	[43]
9	Ti-O-Ti vibration	TiO_2_	450–700	-	615	612	610	Confirms presence of TiO_2_ nanoparticles	[35]

**Table 4:** XRD comparison of the experimental XRD peak positions with the standard JCPDS data for TiO_2_ nanoparticles.

Experimental 2θ (°)	(hkl)	JCPDS Card No.
25.3	(101)	21-1272
37.8	(004)	21-1272
48.0	(200)	21-1272

**Table 5:** Thermal stability parameters of developed blend HFMs.

Membrane	T_5_ (°C)	T_50_ (°C)	Residual Mass (%)
PP	229.74	609.31	25.378
PPT3	336.41	622.25	35.359
PPT5	348.01	622.31	34.663
PPT7	342.59	625.7	38.3

**Table 6:** Comparative CO_2_/CH_4_ separation performance and fabrication methods of various membranes across a 2–8 bar pressure range.

Materials	Fillers	Geometry/Method	Performance Parameter			Operating Pressure and T = 25 °C				Ref.
				2 bar	3.5 bar	4 bar	5 bar	6 bar	8 bar	
PES/PVAc	-	HFMs Dry–wet phase inversion	Permeance (GPU)	84.32		83.79	-	83.43	83.06	This work
			Ideal Selectivity	22.605		22.707	-	23.369	23.867	
PES/PVAc	TiO_2_ 3 wt.%		Permeance (GPU)	89.41		88.10	-	87.22	84.12	
			Ideal Selectivity	26.143		26.142	-	25.77	24.814	
PES/PVAc	-TiO_2_ 5 wt.%		Permeance (GPU)	94.71		94.03	-	93.95	92.22	
			Ideal Selectivity	27.938		28.407	-	30.112	29.748	
PES/PVAc	TiO_2_ 7 wt.%		Permeance (GPU)	95.97		95.63	-	94.91	94.08	
			Ideal Selectivity	27.187		27.559	-	27.832	27.916	
TPESU		HFMs Dry–wet phase inversion	Permeance (GPU)		85.1					[56]
			Selectivity		34					
PEI/PVAc	TiO_2_	Flat sheet/wet phase inversion	Permeance (GPU)	19.7		19.20	-	18.90	-	[57]
			Selectivity	41.2		38.30	-	39.10	-	
PSF	Fumed silica	HFMs Dry–wet phase inversion	Permeance (GPU)				90.04			[58]
			Selectivity				32.74			
P84/PES blends	-	HFMs/Dry–wet phase inversion	Permeance (GPU)	2.12		-		-	-	[59]
			Selectivity	51.6		-		-	--	
PES/Pebax-1657	ZIF-8	Flat sheet Dry/wet phase inversion	Permeance (GPU)	35.2		39.8		43.1	48.2	[60]
			Selectivity	24.5		28.3		31	32.5	
PSF/Pebax-1657	0.5 wt.% MXene	Thin film composite	Permeance (GPU)	-		20		23.6	24	[61]
			Selectivity	-		22.1		30.2	30.8	

[1 GPU = 1 × 10^−6^ cm^3^ (STP)/cm^2^·s·cm·Hg]; [1 Barrer = 1 × 10^−10^ cm^3^ (STP)·cm/cm^2^·s·cm·Hg].

## Data Availability

The original contributions presented in this study are included in the article. Further inquiries can be directed to the corresponding author.

## Abbreviations

The following abbreviations are used in this manuscript:

PES	Polyether sulfone
PVAc	Polyvinyl acetate
PSF	Polysulfone
PI	Polyimide
GO	Graphene oxide
TiO_2_	Titanium dioxide
CO_2_	Carbon dioxide
MMM	Mixed matrix membranes
HFMs	Hollow fiber membranes
GPU	Gas Permeation Unit
FTIR	Fourier-transform infrared
XRD	X-ray diffraction
DSC	Differential Scanning Calorimetry
TGA	Thermogravimetric Analysis
FESEM	Field Emission Scanning Electron Microscopy
TPESU	poly trimethyl phenylene ethersulfone
